# Effect of Blue Light Filtering Intraocular Lenses on Visual Perception

**DOI:** 10.3390/medicina57060559

**Published:** 2021-06-01

**Authors:** Ivajlo Popov, Denisa Jurenova, Jela Valaskova, Diego Sanchez-Chicharro, Jana Stefanickova, Iveta Waczulikova, Vladimir Krasnik

**Affiliations:** 1 Department of Ophthalmology, Faculty of Medicine, Comenius University, 82101 Bratislava, Slovakia; ivajlo.popov@gmail.com (I.P.); deniska@jurenova.sk (D.J.); jelavalasek@hotmail.com (J.V.); jstefanicka@gmail.com (J.S.); 2 Eye Clinic, Martin University Hospital, 03601 Martin, Slovakia; sandi781@hotmail.com; 3 Faculty of Mathematics, Physics and Informatics, Comenius University, 84248 Bratislava, Slovakia; waczulikova@fmph.uniba.sk

**Keywords:** intraocular lens (IOL), blue filter, yellow-tinted IOL, contrast sensitivity, color vision

## Abstract

*Background and Objectives:* This retrospective consecutive case control study compares best-corrected visual acuity (BCVA), mesopic contrast sensitivity (CS), color vision, and glare between a group of eyes with blue-light-filtering intraocular lenses and another with UV-light-filtering intraocular lenses. *Materials and Methods:* We used Early Treatment Diabetic Retinopathy Study charts to compare BCVA, Rabin charts for mesopic CS testing, Oculus HMC Anomaloscope MR to test for chromatic discrimination, and Oculus Mesotest II to measure scotopic CS with glare. For analysis, we used descriptive statistics and compared means with parametric and non-parametric tests. The level of significance was set as α = 0.05. *Results:* For the group with the blue-light-filtering intraocular lens, the average results were BCVA = 0.96 (SD ± 0.09), CS = 1.78 log (SD ± 0.12), chromatic discrimination results M = 63.91 (SD ± 11.88), R = 60.07 (SD ± 7.89). For mesopic CS with glare, the group achieved on average 2.54 (SD ± 1.50) points out of 4. For the group with the UV-light-filtering intraocular lens, the average results were BCVA = 0.93 (SD ± 0.14), CS = 1.79 log (SD ± 0.13), chromatic discrimination results M = 65.38 (SD ± 17.14), R = 60.79 (SD ± 10.39). For mesopic CS with glare, this group achieved an average of 2.79 (SD ± 1.53) points out of 4. *Conclusion:* No significant differences (*p* > 0.05) were found in any of the tested parameters between the analyzed groups. Slight shift in color vision was observed, although not statistically significant.

## 1. Introduction

Cataract surgery has become a routine surgery to replace the human senescent lens with an artificial intraocular lens (IOL) and to restore clarity to the optical media. The human lens accumulates yellow chromophores, which increase the absorption of short-wavelength visible light [1,2], with aging. As more evidence of short-wavelength retinotoxicity emerged [3,4,5], the blue-light-filtering IOL (BFIOL) started to be more popular from the early 1990s [6]. These lenses were considered to give more protection to the retina than clear (UV-filtering) IOLs. There are still discussions about the potential advantages and disadvantages of BFIOL. Some authors believe their benefits [7,8] are worthwhile and others consider them to be of no importance [9,10].

One of the main possible benefits of the BFIOL is its ability to filter high-energy short-wavelength light and thus protect the retina from damage and possible development of age-related macular degeneration (AMD) [4,11,12]. Other possible benefits are improvement in contrast sensitivity [13,14] and reduced glare and reduction in cyanopsia [15]. Altering color vision [16], scotopic contrast sensitivity [17], and interference with the sleep–wake cycle [18] are considered the main drawbacks of the BFIOL [15]. One of the possible effects of BFIOL on sleep–wake cycle is its reduction in the amount of melatonin [19], which is important for circadian rhythm, but the results are ambiguous [20,21].

In our study, we compared the effect of the blue filter on visual acuity, contrast sensitivity, glare, and color vision.

## 2. Methods

The design was for a consecutive case–control study at a single university hospital (Department of Ophthalmology, Faculty of Medicine, Comenius University and University Hospital Ruzinov, Bratislava). The aim of this study was to compare visual performance between the groups with implanted BFIOL and those with clear UV-blocking IOL (UVIOL). The study was approved by the local ethics committee and adhered to the tenets of the Declaration of Helsinki.

The patients were recruited according to the following specified criteria. Eligible subjects were selected from the list of patients who underwent cataract surgeries in the year 2013, so all subjects had undergone cataract surgery on both eyes in the University Hospital, Ruzinov at least one year before further examination. A one-year period was chosen to ensure that no postoperative condition like corneal edema, wound healing, or neuroadaptation would interfere with the examination and the results. Eyes with BFIOL were implanted with BioLine Yellow Accurate Aspheric IOL (i-Medical, Mannheim, Germany) with wavelength cutoff values of 10% transmittance at 390 nm and blue-light filter for 430 nm wavelength. The UVIOL group was implanted with IOLs from different manufacturers, TECNIS ZCB00 (Johnson & Johnson, New Brunswick, NJ, USA) and Softec HD (Lenstec, St Petersburg, FL, USA), with wavelength cutoff values of 10% transmittance at 377, 7 nm [22] and 390 nm, respectively). All patients who had undergone eye surgeries other than uncomplicated cataract surgery were excluded. Past records of selected patients were checked for any note of macular degeneration, glaucoma, diabetic retinopathy, chemotherapy for oncological diseases, and other eye diseases which could cause bias in further analysis. Eligible patients were added to the sample (118 patients, 236 eyes—124 BFIOL and 112 UVIOL). Selected patients were invited for examination by phone or mail. Only 67 patients accepted the invitation for the examination. At the beginning of the examination, the medical history of all patients was double-checked for any above-mentioned conditions. The patients’ informed consent was signed on the day of the examination. All patients had the same group of IOL implanted in both eyes. Examinations took place from November 2015 to December 2016.

### 2.1. Examination Protocol and Data Collection

Best-corrected visual acuity (BCVA) and best correction were examined first on ETDRS charts. Contrast sensitivity (CS) was tested with the best correction on the Rabin chart for the ETDRS cabinet. Both BCVA and CS were measured in photopic light conditions at 85 cd/m^2^. BCVA and CS were measured monocularly. The minimum BCVA required to proceed to the next examination was set at 0.7 and the minimum CS score was set at 1.50 log CS. If the result of any of these examinations was lower, we proceeded directly to dilated fundus examination to double-check recruitment exclusion criteria or to find a possible cause of the impaired visual performance. If the cause was posterior capsule opacifications, we performed a YAG posterior capsulotomy and repeated complete examination after 3 weeks.

After successful examination of BCVA and CS, we proceeded to test color vision by screening for inherited red–green color blindness with Ishihara plates. Chromatic discrimination changes in the blue–green spectrum were tested with anomaloscope (Oculus, HMC Anomaloskop MR Moreland and Rayleigh, type 47700). We used a manual Moreland blue–green test with neutral adaptation in the device setting. The mixed color field (M axis) can be adjusted by the patient from 0 to 100 (100, for example, results in a setting of the mixed color field without the color blue). The comparison field (R axis) can be adjusted in brightness from 0 to 100. Neutral adaptation is achieved by presenting a white fluorescent field, which is comparable to the standard light source C (6770° Kelvin). This field was repeatedly presented every three seconds. After the testing procedure was explained and a trial run was performed, the patients were tested monocularly with the best distance correction. 

The glare was tested at the end as there was the necessity for dark adaptation. After dark adaptation (at least 15 min), we tested glare and scotopic CS with the device designed for this task (Mesotest II, Oculus GmbH). The test with this device was performed in the presence and in the absence of glare. Landolt C was tested in four levels of contrast. The Landolt ring positions were randomly placed. Moving to the next level with lower contrast was done when at least three of five positions were successfully recognized. Each correct answer was determined as one point. The maximum point score for the test without glare was four. The maximum score for the test with glare was also four points.

**Figure 1:**
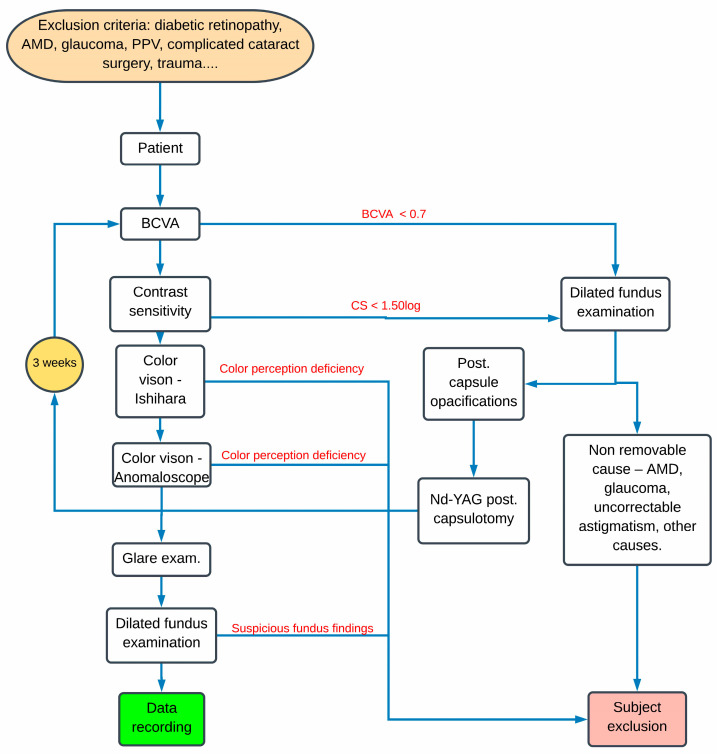
Algorithm of the examination. BCVA—Best Corrected Visual Acuity; CS—Contrast Sensitivity; PPV—Pars Plana Vitrectomy; AMD—Age-related Macular Degeneration.

After all the above examinations, dilated fundus examination was performed on each subject to find any ocular pathologies, especially macular degenerations. Patients with any suspicious findings on the ocular fundus were excluded. Some tests were not completed by all patients. Many patients considered the tasks on the anomaloscope and the Mesotest II as very complicated and refused to be tested on those devices. All BFIOL eyes group were implanted with BioLine Yellow Accurate Aspheric IOL (i-Medical). The UVIOL group was implanted with IOL from two different manufacturers (TECNIS ZCB00 (Johnson & Johnson) and Softec HD (Lenstec) IOL). The full algorithm of examination is described in Figure 1.

### 2.2. Statistical Analysis

The patients’ data were analyzed by means of descriptive and inferential statistics and interpreted in the context of recent knowledge and previous studies. Continuous or interval-scaled variables were first checked for normality by means of visual inspection and the Shapiro–Wilk test. Descriptive and univariate analyses were performed on all selected patients’ characteristics. For normally distributed data, we used parametric tests (equivalent with nonpaired *t*-test in case of two groups) and the Mann–Whitney test for nonparametric analysis. Multiple linear regression for continuous outcomes and logistic regression for categoric/binary outcomes was used to test the effect of demographic variables on examined parameters. *p* values less than 0.05 were considered statistically significant.

## 3. Results

From 67 subjects only 60 were eligible (see above exclusion criteria) for further analysis. Only right eyes were added to the sample for the final analysis. The subjects were made up of 21 males and 39 females. In the female group, there were 16 eyes with UVIOL and 23 eyes with BFIOL. In the male group, there were 12 eyes with UVIOL and 9 eyes with BFIOL. Differences in counts between IOL groups were not statistically significant (*p* = 0.284). 

Descriptive statistics of the analyzed parameters with the *p* values are shown in Table 1.

A two-sample data comparison of the means did not detect significant differences between the means of the analyzed parameters. To address the possibility that the effect of lenses on the analyzed parameters might have been suppressed by age and sex, so we performed multivariable analysis. Multivariable adjustment for age and sex did not change the results, so the results of the univariable analysis can be retained. Table 1 also shows *p* values for the t-test comparing means in analyzed parameters between UVIOL and BFIOL group. No statistically significant differences were found in any of the analyzed parameters.

**Figure 2:**
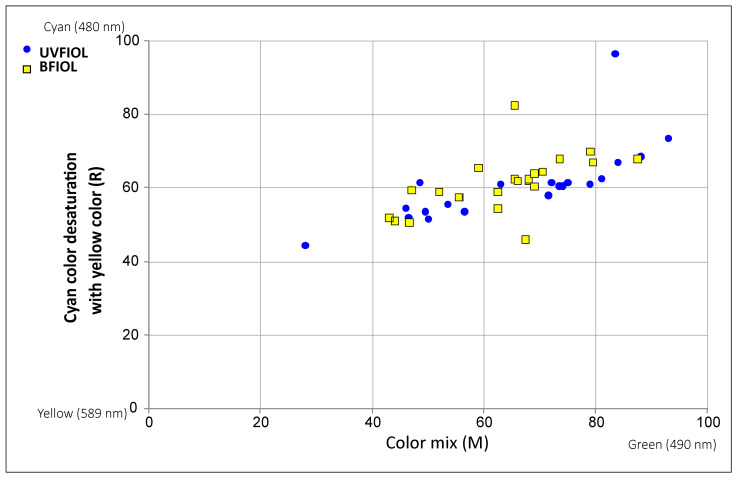
Results from anomaloscope testing. UVIOL—UV blocking intraocular lens; BFIOL—Blue light-filtering intraocular lens.

Results from anomaloscope testing are depicted in Figure 2 for each patient. 

We could notice a slight shift of the BFIOL group towards green and yellow color. However, this shift was not statistically significant (*p* > 0.05).

## 4. Discussion

Many studies have been published to compare the effect of the blue-light-filtering IOL on visual performance [23], but a lot of them lack high precision and repeatability of measurements [24]. In our study, we used devices that provided very stable environments for conducting the visual tests. This ensures our data can be easily compared to other studies with the same devices. To our knowledge, there are only three publications with similar strict examination criteria and methods [25,26,27]. Only two of these studies used an anomaloscope for the Moreland test [25,26] and only one of them used both an anomaloscope for the Moreland test and a Mesotest II [26]. The Khokhars study used a Raylight red–green test on the anomaloscope, in which changes in color vision in the blue spectrum could be overlooked [27]. 

Our study did not find any statistically significant differences in BCVA and scotopic contrast sensitivity. Our findings confirm those of many other studies [10,26,28,29], but they are in contrast with the findings of Wirtitsch, who found lower CS in BFIOL [25]. In Yuan’s study, BFIOL showed even better contrast sensitivity in photopic and mesopic light conditions than UVIOL [8]. 

Contrast sensitivity decreases with age [26,30]. Both groups in our sample had very similar mean ages, which eliminated possible bias. The effect of age was not statistically significant in the multivariable analysis (*p* > 0.05). Low BCVA and CS scores are usually caused by age-related macular degeneration or posterior capsule opacification (PCO). According to Khan, a few small drusen are present in more than 80% of the population between 18 and 53 years [31]. As the age of our subjects was considerably higher, we decided to include subjects with less than three small hard drusen on the fundus on dilated fundus examination. Cases with macular degeneration were excluded from the analysis. For cases with PCO, we performed YAG posterior capsulotomy and repeated the complete examination after three weeks.

We assumed there could be some subtle shift in chromatic discrimination in the blue part of the spectrum because BFIOL blocks the light of a short wavelength. Therefore, we tested chromatic discrimination with an anomaloscope in the Moreland blue–green test. Our study did not yield a statistically significant difference in blue light discrimination. However, in Figure 2, we can observe a slight shift of BFIOL from the blue color, although it is not statistically significant. Additionally, da Costa noticed a higher tritan threshold in the Cambridge color test with blue-light-blocking lenses [32]. However, our findings in this regard were not statistically significant and they are in conformity with the findings of Wirtitsch [25], Muftuoglu [26], and other studies [33,34].

The device, Mesotest II (Oculus GmbH), was used to test scotopic contrast sensitivity with and without glare. One of the possible advantages of BFIOL is the ability to reduce glare [35,36]. In contrast to this assumption, we found even lower scotopic CS in the BFIOL group during the examination with glare. However, those differences were not statistically significant (Table 1). Those findings are similar to those of Muftuoglu [26]. There was no statistically significant difference in scotopic contrast sensitivity without glare.

Our sample of 60 patients is similar to the samples of other studies [15,23], although the sample size of our study could be a limitation in substantiating subtle differences between the groups. We did not test mesopic contrast sensitivity, and if any changes in mesopic contrast sensitivity were present, they could be overlooked. Our study was a retrospective case–control comparative study, which might also be a limitation.

In conclusion, our study found that blue filter IOL showed no statistically significant difference to UV blocking IOL in BCVA, photopic and scotopic contrast sensitivity, color perception, and glare. 

## Figures and Tables

**Table 1:** Descriptive statistics of the analyzed parameters with the *p* values.

		Age (Years)	BCVA	CS (logCS)	Anomaloscope M	Anomaloscope R	Glare−	Glare +
**Total**	*n*	60	60	59	48	48	51	50
	Mean	75.62	0.94	1.78	64.55	60.39	3.96	2.66
	SD	5.48	0.12	0.13	14.27	8.97	0.20	1.51
	Range	63–87	0.70–1.2	1.50–2.0	13–93	44.5–96.5	3–4	0–4
**UVIOL**	*n*	28	28	28	21	21	25	24
	Mean	76.21	0.93	1.79	65.38	60.79	3.92	2.79
	SD	5.53	0.14	0.13	17.14	10.39	0.28	1.53
	Range	63–87	0.70–1.2	1.50–2.0	28–93	44.5–96.5	3–4	0–4
**BFIOL**	*n*	32	32	31	27	27	26	26
	Mean	75.09	0.96	1.78	63.91	60.07	4.00	2.54
	SD	5.47	0.09	0.12	11.88	7.89	0.00	1.50
	Range	67–86	0.70–1.2	1.5–2.0	43–87.5	46–82.5	4–4	0–4
**UVIOL vs. BFIOL**	*p* value	0.434	0.379	0.645	0.738	0.788	0.235	0.549

BCVA—Best Corrected Visual Acuity; UVIOL—UV blocking intraocular lens; BFIOL—Blue light filtering intraocular lens; CS—Contrast sensitivity; Glare +/− Scotopic contrast sensitivity with/without glare.

## Data Availability

The data presented in this study are available on request from the corresponding author. The data are not publicly available due to restriction in sharing of personal data.
